# Supplementary figures and images for: Norovirus infection results in eIF2α independent host translation shut-off and remodels the G3BP1 interactome evading stress granule formation
Source: PLoS Pathog. 2020 Jan 6;16(1):e1008250. doi: 10.1371/journal.ppat.1008250 (PMC6964919; doi:10.1371/journal.ppat.1008250)

**Supplementary Figure 1**

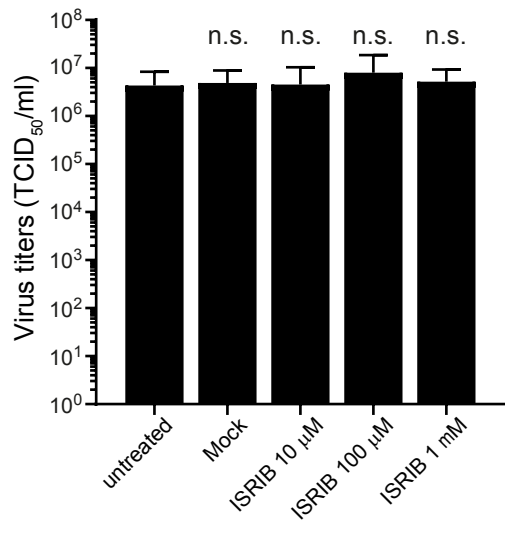

Supplement: S1 Fig — Bar plots of the viral titres measured by TCID50 (logarithmic scale) from BV2 cells inoculated with MNV (MOI 1) for 16h in presence of increasing doses of ISRIB from 10nM to 1μM. Mean ±SD (n = 3), statistical analysis given above the bars, n.s, not significant. (PDF) [file ppat.1008250.s002.pdf]

Supplementary Figure 2

A

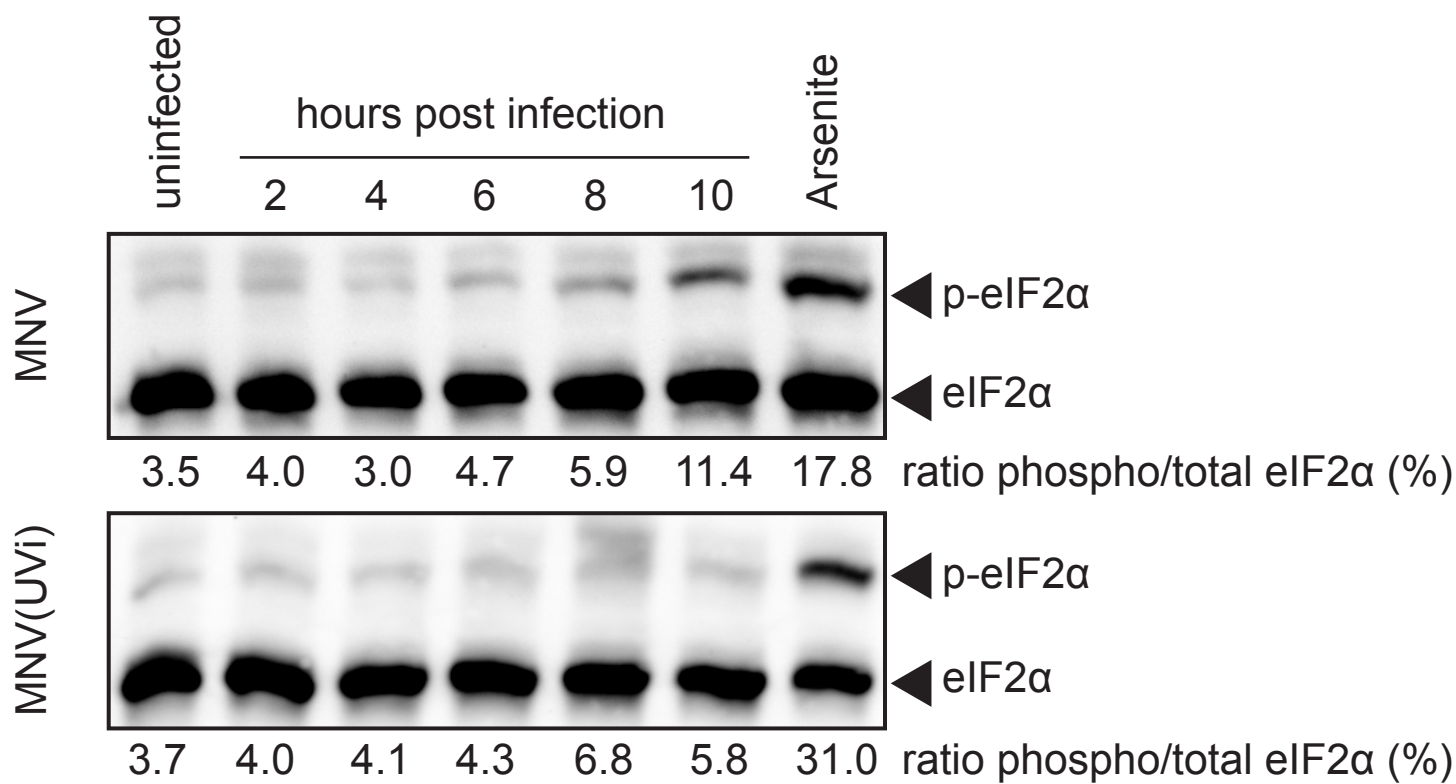

B

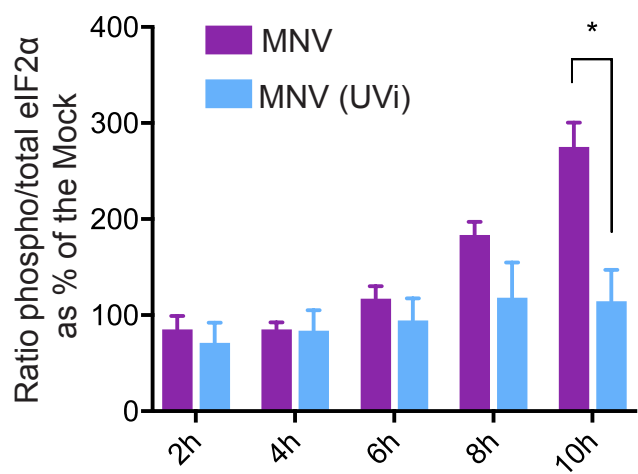

Supplement: S2 Fig — (A) Representative PhosTag acrylamide gel analysis (n = 3) of RAW264.7 cells infected (MOI 10) for the indicated times with replicative MNV (upper panel) or non-replicative virus MNV(UV) (lower panel). Naive cells (Mock) were cultivated in parallel for 10h and arsenite-treated cells were used as control. Lower band, unphosphorylated P-eIF2α, upper band, P-eIF2α. The percentages of P-eIF2α compared to total eIF2α for each sample are given below the blots and plotted in (B) total eIF2α being the sum of the signal intensity of the lower and upper bands in each lane. (PDF) [file ppat.1008250.s003.pdf]

Supplementary Figure 3

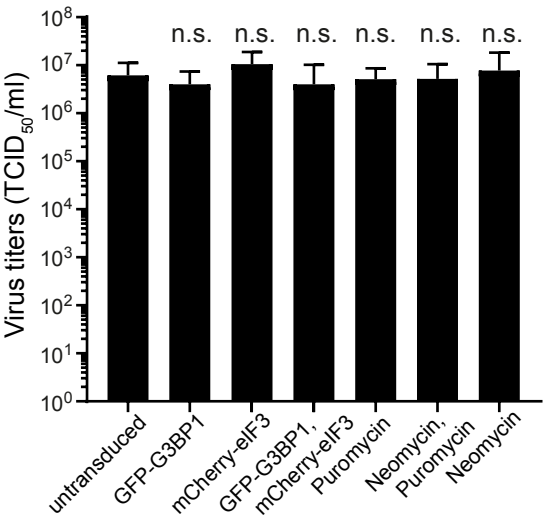

Supplement: S3 Fig — (A) Bar plots of the viral titres measured by TCID50 (logarithmic scale) from BV2 cells w.t., Puro, Neo, Puro-Neo, GFP-G3BP1, mCherry-eIF3E and GFP-G3BP1/mCherry-eIF3E inoculated with MNV (MOI 1) for 16h. Mean ±SD (n = 3), statistical analysis given above the bars, n.s, not significant. BV2 GFP-G3BP1 cells were infected with MNV for 9hp.i prior fixation. (B) Representative view of confocal analysis (n = 2) of GFP-G3BP1 subcellular localisation with immunodetection of G3BP1 (magenta) and MNV NS3 (gold). Scale bars, 10μm. (PDF) [file ppat.1008250.s004.pdf]

Supplementary Figure 4

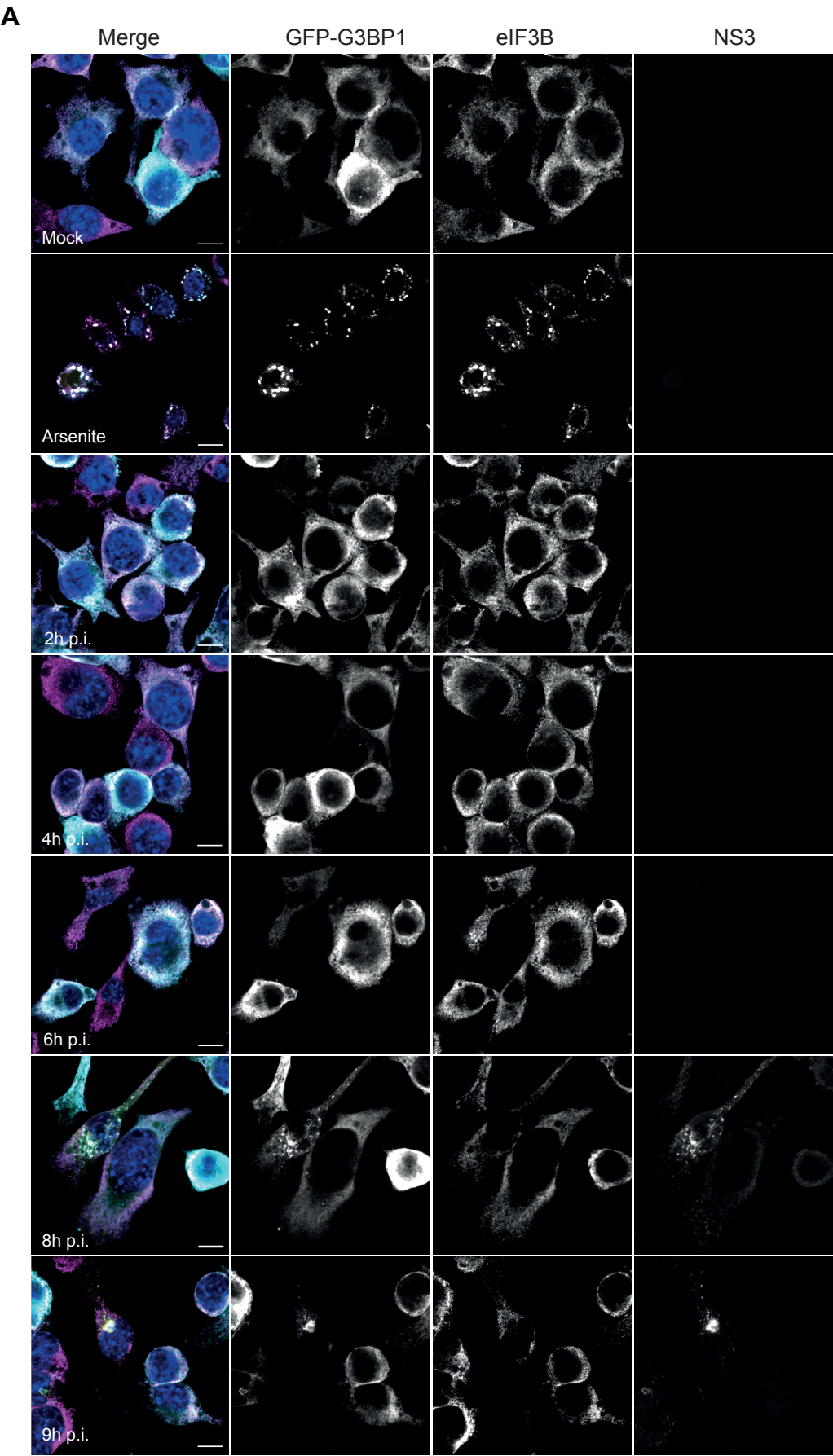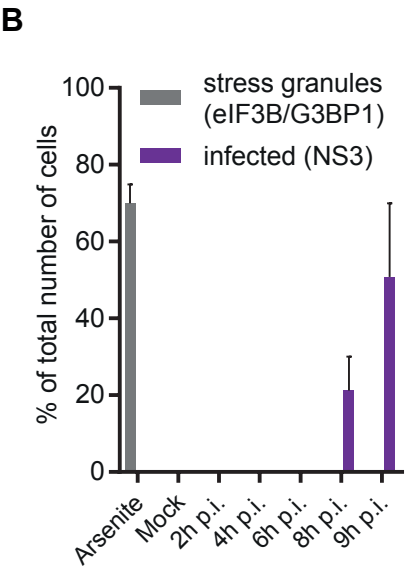

Supplement: S4 Fig — Cells cultures infected with MNV do not display formation of SG in time course experiments (A and B). MNV-infected BV2 GFP-G3BP1 (MOI 10) were fixed at 2, 4, 6, 8 and 9h p.i. Cells treated with 0.1mM of arsenite for 45min were used as a positive control and both mock- and arsenite-treated cells were grown alongside the MNV-infected cells and fixed at 9h p.i. (A) Representative view (n = 2) of a confocal analysis of the formation of SG by dual detection of GFP-G3BP1 (cyan) and eIF3B by immunofluorescence (magenta). The efficiency of MNV infection and replication was addressed by immunodetection against MNV NS3 (gold). Nuclei were stained with DAPI. Scale bars, 10μm. (B) Bar plot of the percentage of cells displaying SG (GFP-G3BP1 and eIF3B positive foci, grey bars) and MNV-infected cells (NS3 positive, magenta bars), mean ± SD for 100 GFP-positive cells analysed across at least 10 acquisitions. (PDF) [file ppat.1008250.s005.pdf]

**Supplementary Figure 5**

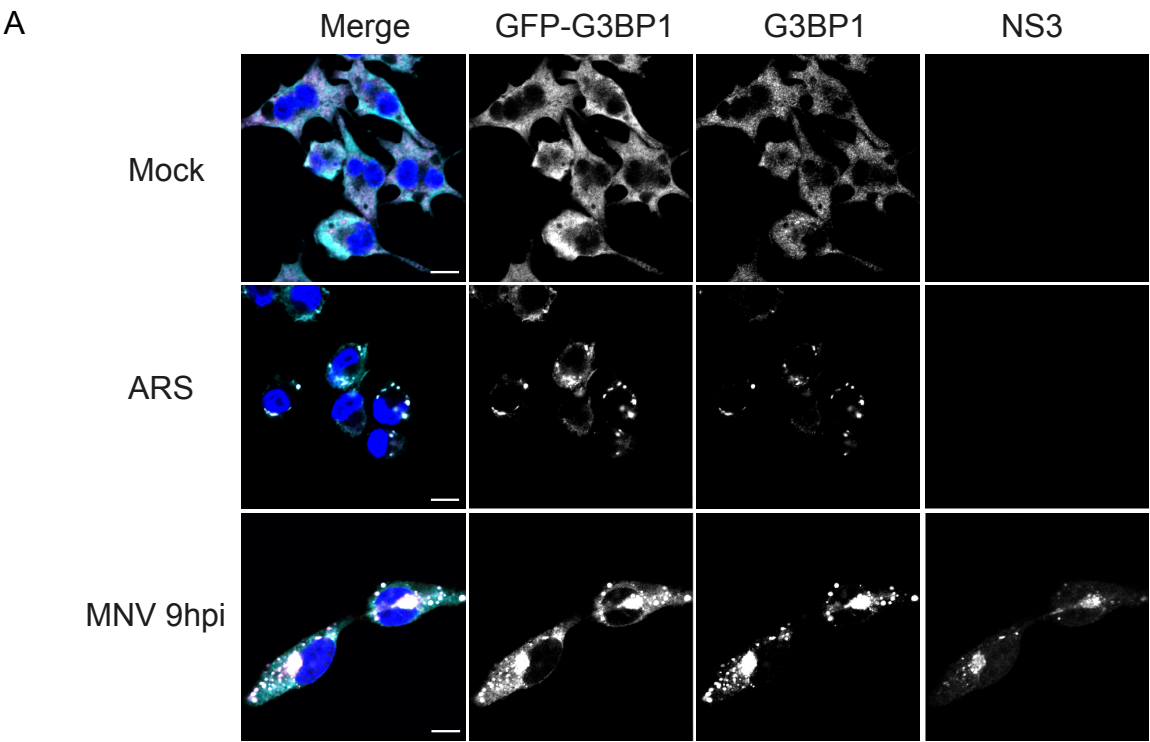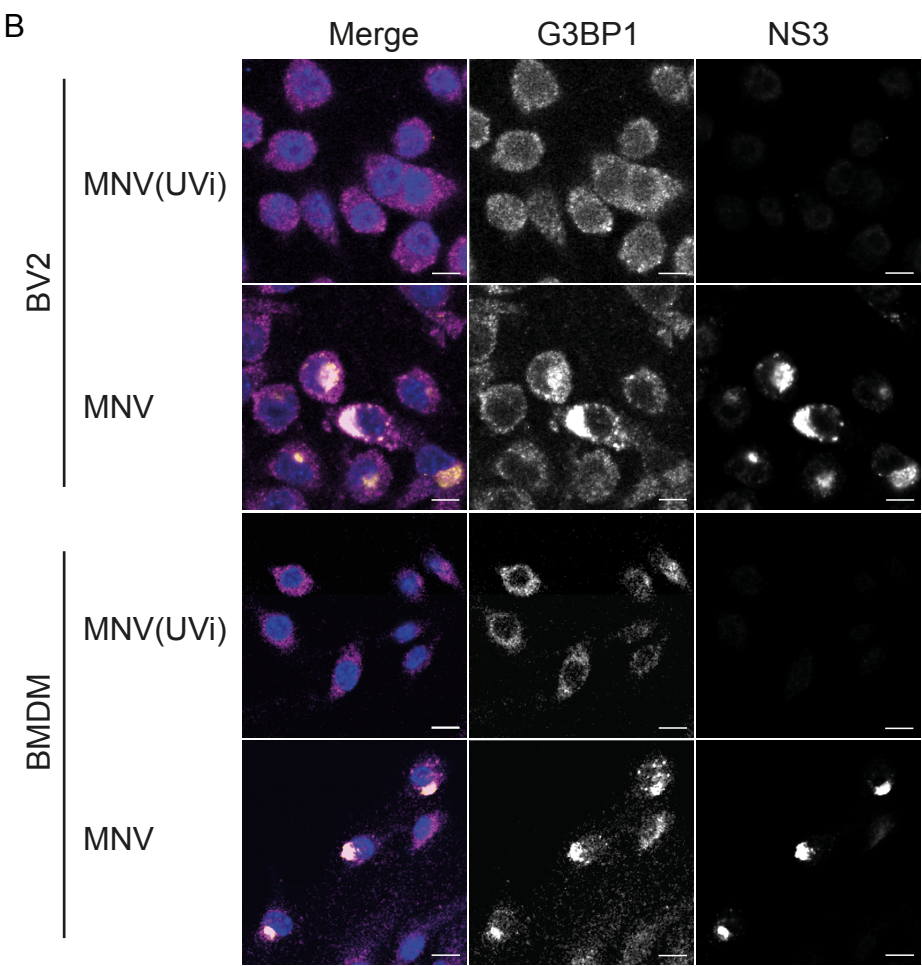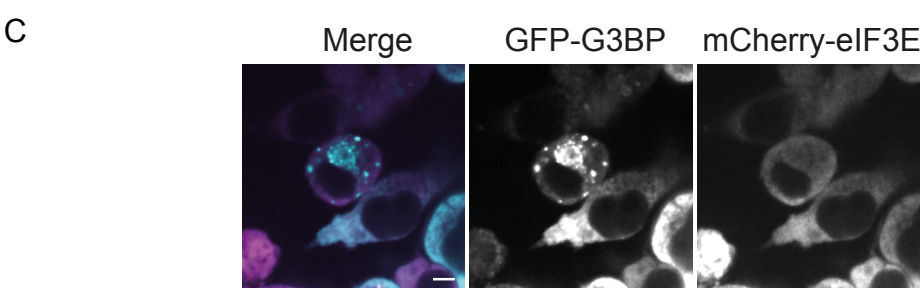

Supplement: S5 Fig — Endogenous G3BP1 colocalises in vivo with NS3 in MNV-infected cells (A and B). (A) MNV-infected BV2 GFP-G3BP1 (MOI 10) were fixed at 9h p.i. Cells treated with 0.1mM of arsenite for 45min were used as a positive control and both mock- and arsenite-treated cells were grown alongside the MNV-infected cells and fixed at 9h p.i. Representative view of confocal analysis (n = 2) of GFP-G3BP1 subcellular localisation (cyan) with immunodetection of G3BP1 (magenta) and MNV NS3 (gold). Nuclei were stained with DAPI. Scale bars, 10μm. (B) MNV(UV)- or MNV-infected BV2 or BMDM were incubated respectively for 9 and 15h p.i prior fixation. Representative view (n = 3) of a confocal analysis of the subcellular distribution of G3BP1 (magenta), showing MNV replication complexes identified by immunodetection against MNV NS3 (gold). Nuclei were stained with DAPI. Scale bars, 10μm. MNV-induced G3BP1 aggregation is observed in living cells (C). Representative view of a time-lapse acquisition by confocal microscopy of BV2 cells expressing GFP-G3BP1 (cyan) and mCherry-eIF3E (magenta) in culture infected with MNV (MOI 20) at 10h15 p.i. Scale bar, 5μm. (PDF) [file ppat.1008250.s006.pdf]

Supplementary Figure 6

A

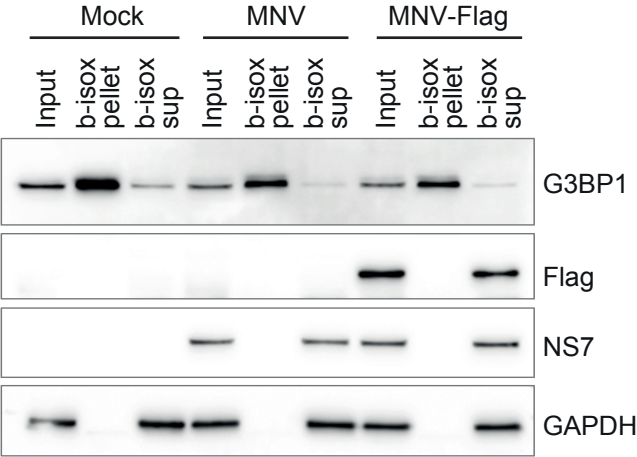

B

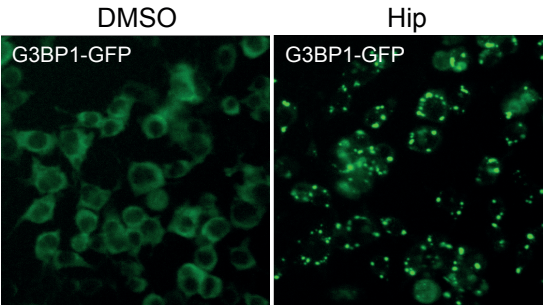

C

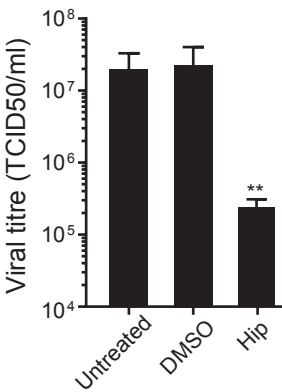

Supplement: S6 Fig — BV2 GFP-G3BP1 cells were treated with 1μM of hippuristanol or DMSO for 1h prior inoculation with MNV (MOI 1) for 16h (B and C). (B) Representative view (n = 3) of the induction of SG formation in hippuristanol treated cells (Hip) by fluorescence microscopy. (C) Bar plots of the viral titre measured by TCID50 (logarithmic scale) from BV2 GFP-G3BP1 untreated, treated with 1μM of hippuristanol (Hip) or DMSO for 1h prior inoculation with MNV (MOI 1) for 16h. Mean ±SD (n = 3), statistical analysis given above the bars, ** P < 0.05. (PDF) [file ppat.1008250.s007.pdf]

## Supplementary Figure 7

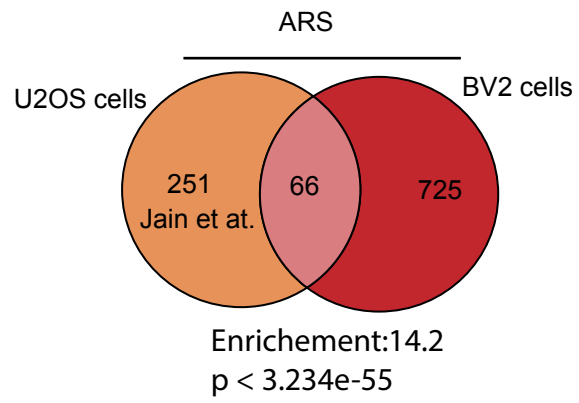

Supplement: S7 Fig — Venn diagram of the SGs interactome showing the common elements between human cells (U2OS cells) and mouse cells (BV2 cells). The hypergeometric p-value and enrichment factor are displayed. (PDF) [file ppat.1008250.s008.pdf]

Supplementary Figure 9

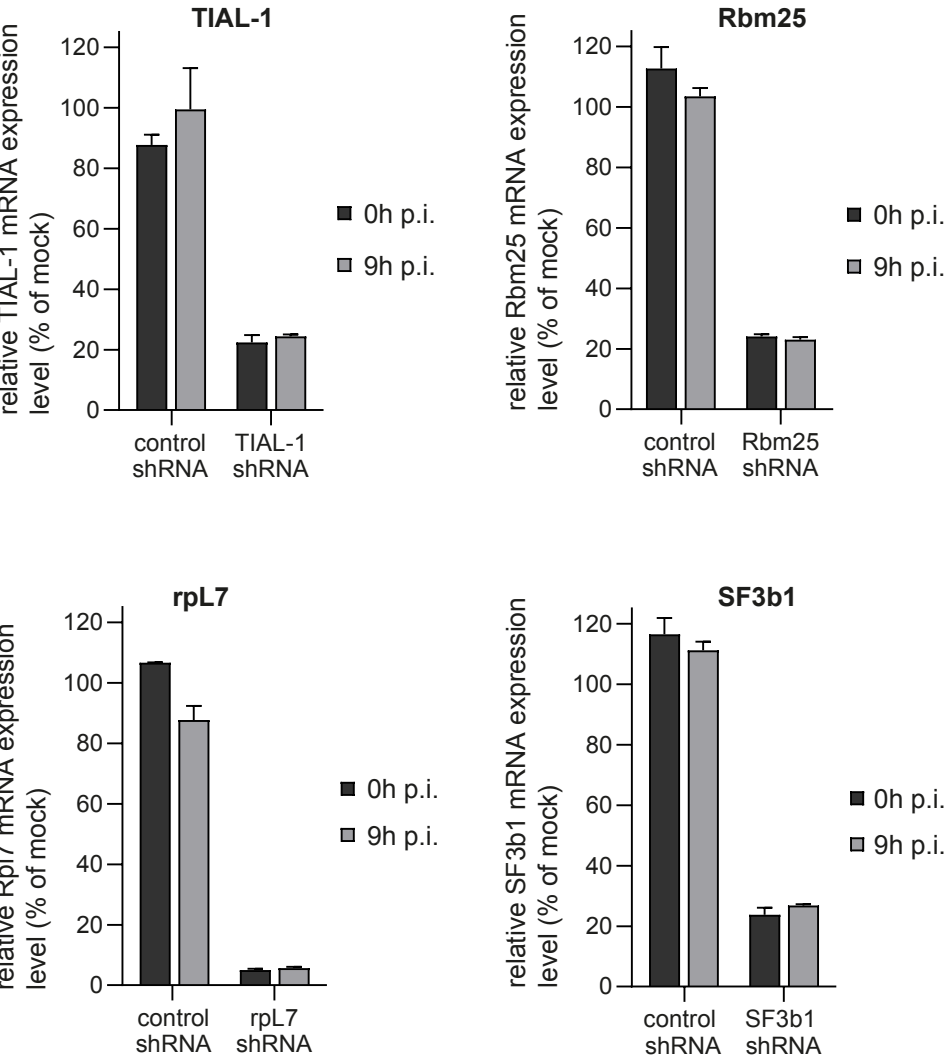

Supplement: S9 Fig — Transient silencing via lentiviral particles transduction of shRNA. 1.25x10^5 BV2 cells were inoculated with10^8 pU of lentiviral particles for 48 and 57h before total RNA extraction. Representative (average of qPCR repeat ±SD, n = 3) efficiencies of silencing measured by qRT-PCR are shown as the level of the mRNA of interest normalised by the level of GAPDH mRNA and presented as percentage of the control shRNA in cells silenced for TIAL-1, RBM25, RPL7 and SF3B1. (PDF) [file ppat.1008250.s010.pdf]
