# Supplementary material for: Norovirus infection results in eIF2α independent host translation shut-off and remodels the G3BP1 interactome evading stress granule formation
Source: PLoS Pathog. 2020 Jan 6;16(1):e1008250. doi: 10.1371/journal.ppat.1008250 (PMC6964919; doi:10.1371/journal.ppat.1008250)

Supplementary Figure 8

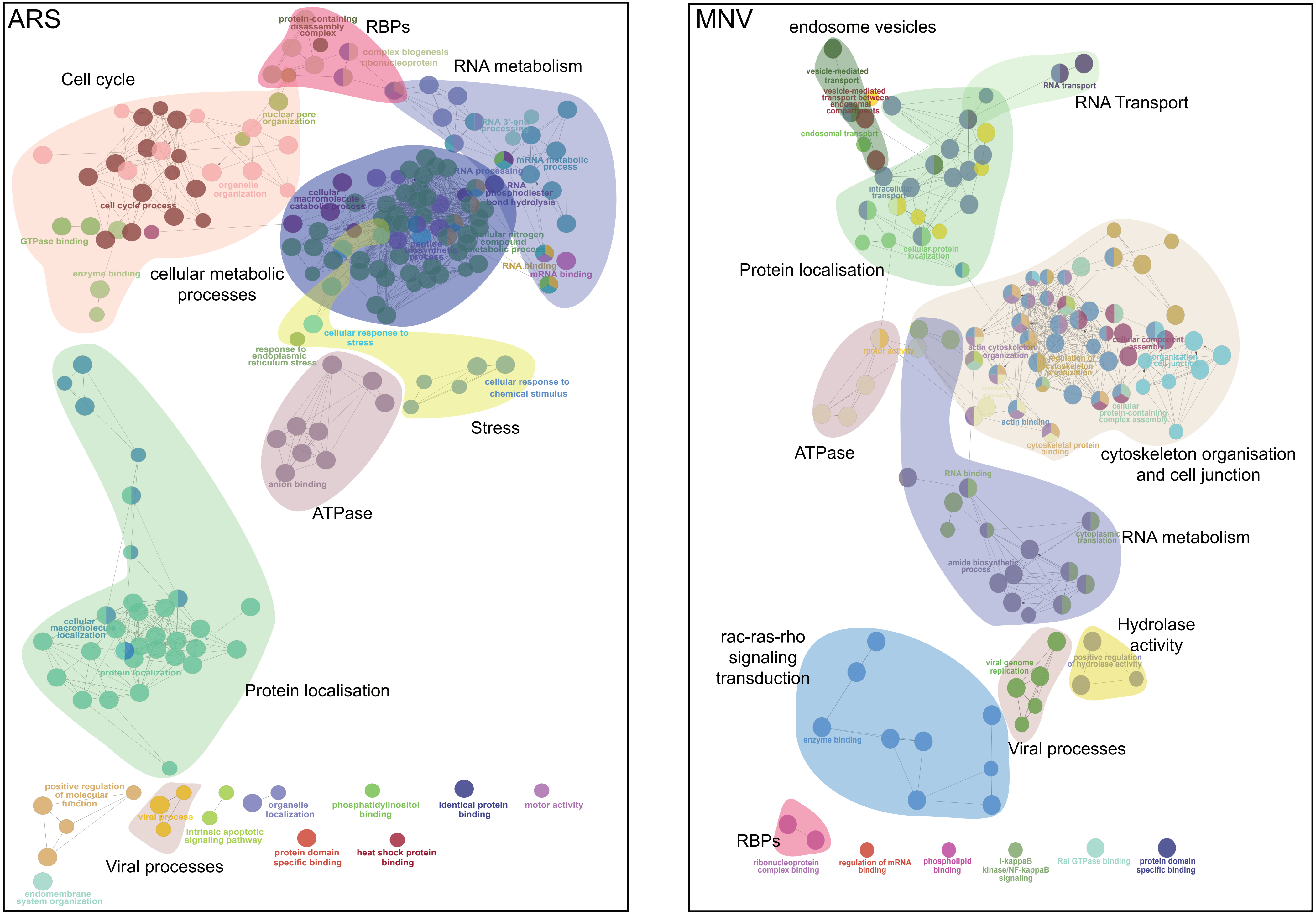

Supplement: S8 Fig — Cytoscape clustering was performed using ClueGO app based on GO terms (molecular function and biological process) considering two side hypergeometric test and Bonferroni correction p-value<0.005, using GO term fusion and layout “perfused force direct” for a clear representation. The nodes were grouped accordingly to GO terms and the node size corresponds to the significance of each GO term in the network. We arbitrarily added an additional colour pattern to the cluster in order to highlight the difference between the two groups on analysis (ARS and MNV). (PDF) [file ppat.1008250.s009.pdf]
